# Biochar and milk vetch synergistically enhance rice yield and soil fertility via regulating N-cycling in reddish paddy fields

**DOI:** 10.3389/fpls.2026.1839609

**Published:** 2026-06-19

**Authors:** Jiahui Zhou, Kun Zhang, Farooq Shah, Su Shiung Lam, Zhijian Xie

**Affiliations:** 1 College of Land Resources and Environment, Jiangxi Agricultural University, Nanchang, China; 2 Jiangxi Institute of Red Soil and Germplasm Resources, Nanchang, China; 3 Department of Agronomy, Abdul Wali Khan University Mardan, Khyber, Pakhtunkhwa, Pakistan; 4 Higher Institution Centre of Excellence (HICoE), Institute of Tropical Aquaculture and Fisheries (AKUATROP), Universiti Malaysia Terengganu, Kuala Nerus, Terengganu, Malaysia; 5 University Centre for Research and Development, Department of Chemistry, Chandigarh University, Mohali, Punjab, India

**Keywords:** microbial functions, milk vetch, rice productivity, rice straw biochar, soil N availability

## Abstract

**Introduction:**

Biochar and milk vetch (MV) are known to improve soil fertility and crop yield. However, the effects of the combined application of rice straw biochar (RSB) and MV over multiple years on nitrogen (N) dynamics, microbial ecology, and rice yield remain unclear. This study is to evaluate the synergistic effects of RSB and MV over multiple growing seasons.

**Methods:**

A three-year field experiment was conducted with four treatments, N fertilizer alone (CK), N+RSB (B), N+MV (M), and N+RSB+MV (BM), and measured various indicators of soil health and rice yield.

**Results:**

Soil organic C, total N, inorganic N (NH_4_^+^ and NO_3_^−^), microbial biomass C/N, and enzyme activities (urease, nitrate, and nitrite reductase) were 14%–30%, 12%–25%, 18%–40%, 20%–45%, and 15%–50% higher in the B and M treatments than in the CK; the greatest increases in these parameters (by 14%–89%) were observed in the BM treatment. Microbial diversity (α-diversity of soil microbes) was increased and the expression of N-cycling functional genes (*nif*H, *amo*A, *nar*G, *nir*S/*nir*K, *nos*Z) was 9.1%–400% higher in the BM treatment than in the CK. High gene abundances (AOA *amo*A, AOB *amo*A, *nar*G, *nir*S, *nir*K, and *nos*Z) were associated with increased N uptake and grain yield. Microbial biomass C and N were key drivers of shifts in microbial communities influencing N-cycling genes. Increased soil C and N availability in the BM treatment stemmed from changes in the expression of microbial genes and enzyme activities enhancing N assimilation and rice yield.

**Discussion:**

Hence, combining RSB with MV can promote soil fertility, rice productivity and ecological benefits.

## 1 Introduction

Rice (*Oryza sativa L.*) is a staple food for over half of the global population and plays a critical role in ensuring global food security (Yuan et al., 2021). In particular, China is a leading rice producer, contributing approximately 28% of the world’s total rice output (Su et al., 2023). Nitrogen (N) availability is a major determinant of rice yield, accounting for more than 60% of total productivity (Ma et al., 2023). However, the excessive input of N fertilizer has led to soil degradation, N losses, and reduced production efficiency (Kamran et al., 2021). Therefore, optimizing N management practices is essential for stable and sustainable rice production. China produces approximately 200 million t·yr^-1^ of rice straw annually, applied as a nutrient-rich amendment for crops (Nan et al., 2020). However, challenges include slow decomposition, N competition with crops, increased risk of pests and diseases, soil acidification, and greenhouse gas (GHG) emissions (Wang et al., 2019).

Rice straw biochar (RSB) is extensively used to enhance soil fertility. Recent studies have highlighted its key role in enhancing N uptake, utilization, and crop productivity (Dai et al., 2024). These positive effects of RSB stem from its highly porous structure, providing habitats for microbes, thus promoting enzyme activities and microbial biomass (Zhou et al., 2019b). RSB has positive effects on soil inorganic N, functional genes of nitrifiers and denitrifiers, and the *nir*S/(*nir*K+*nir*S) ratio (Xiao et al., 2019). However, RSB with a high C/N ratio might lead to short-term N immobilization, reducing N availability to crops. Given that most studies have focused on single and high-dose applications (≥ 20 t·ha^-1^) of RSB over short time scales, much remains to be learned regarding its effects on crops.

The use of milk vetch (*A. sinicus L*., MV) as a green manure (GM) has received increased attention for its efficient use of agricultural resources (e.g., water, light) in winter and its ability to enhance crop productivity, soil fertility, and microbial functions (Xia et al., 2025). MV can be used to develop sustainable strategies that simultaneously increase rice yields and reduce GHG emissions (Yang et al., 2023). Gao et al. (2021) revealed that MV modulate the structure of N-cycling microbes. Therefore, incorporating MV into rice-based cropping systems increases N availability as well as improves microbial functions, regulating N-cycling processes. Moreover, the application of MV and N fertilizer or straw increases the abundance of functional genes related to organic N mineralization, denitrification, and fixation (e.g., *ghd*A, *nir*K/S, *nif*H), while reducing nitrifiers (e.g., AOB *amo*A) (Zhou et al., 2023). Previous studies indicate that MV increases the abundances of N-cycling microbes, such as *Bacteroidetes* and *Proteobacteria*, and decreases *Acidobacteria* and *Chloroflexi* (Xia et al., 2023); the co-application of GM and straw alleviates limitations associated with the sole application of straw (N-fixation) or GM (active N losses) (Li et al., 2025). The combined application of RSB and MV is considered to generate complementary effects due to their contrasting C/N characteristics (Zhou et al., 2019a). While RSB provides a stable C source with slow nutrient release, MV rapidly mineralizes and supplies available N (Cheng et al., 2023). Therefore, ensuring the appropriate use of RSB’s high C and MV’s high N content to enhance N uptake in crops remains a challenge for achieving sustainable rice production.

Recently, the co-application of MV and RSB has garnered widespread attention for improving rice yield and soil microecology (Xie et al., 2022; Liang et al., 2025; Liu et al., 2026). However, most previous studies have focused on short-term, single-amendment applications, often at high biochar doses, and have not addressed the multiple-year, field-scale impacts under realistic agronomic management. Elucidating these temporal dynamics is crucial, as prolonged biological restructuring transforms transient N availability into a stabilized, highly efficient N-cycling network. Ultimately, this fundamental shift drives sustained enhancements in N assimilation and rice yield that remain undetectable within a single growing season.

To address this knowledge gap, we conducted a three-year field experiment in reddish paddy fields to: (i) assess the effects of co-applying RSB and MV on soil C and N status, N uptake, and rice yield; (ii) characterize changes in soil microbial structure, diversity, enzyme activities, and functional genes related to N-cycling; and (iii) elucidate the mechanistic linkages between soil N-cycling processes and rice productivity under combined amendment. This study is the first to comprehensively evaluate the synergistic impacts of RSB and MV in reddish paddy fields over multiple growing seasons. By integrating biochemical, microbiological, and agronomic analyses, it provides novel mechanistic insights into how these amendments jointly regulate soil N-cycling and enhance yield. The findings offer a practical, sustainable strategy for recycling crop residues and green manure to improve soil fertility, increase productivity, and deliver eco-environmental benefits in rice-based systems.

## 2 Materials and methods

### 2.1 Experimental site, soil, and climate

This field experiment was conducted at the Jiangxi Institute of Red Soil and Germplasm Resources in Jinxian, Jiangxi Province, China (28°20′54″N, 116°16′17″E, **Supplementary Figure 1**). This region has a subtropical monsoon humid climate with an annual average temperature of 18.1°C, an annual average sunshine duration of 1950 h, and an annual mean precipitation of 1632 mm. The initial chemical properties of topsoil were: pH 5.23 (1:2.5, soil: H_2_O), soil organic carbon (SOC) 19.3 g·kg^-1^, total N (TN) 1.51 g·kg^-1^, available N (AN) 176.9 mg·kg^-1^, available P (AP) 31.6 mg·kg^-1^, and available potassium (AK) 75.7 mg·kg^-1^.

### 2.2 Milk vetch and rice straw biochar preparation

The MV (*var. Ganzi* No.1) seeds were sown in mid-October without tillage (except in the CK and biochar treatments), and no additional fertilizers were applied in winter regardless of whether the fields were fallow or with MV. The MV included total C (TC) 413.8 g·kg^-1^ and TN 25.3 g·kg^-1^ (with the C/N ratio 16.4), total phosphorus (TP) 1.38 g·kg^-1^ and total potassium (TK) 26.0 g·kg^-1^.

RSB (< 5 mm) was produced from rice straw via pyrolytic carbonization at 450–500°C under oxygen-limited conditions in a self-made auto-ignition furnace. The furnace temperature increased at a rate of 5 °C min^-1^ up to 500 °C, which was maintained for 2 h. RSB was produced every year of the experiment from the preceding year’s harvested rice straw. The properties of RSB were as follows: pH 9.97, TN 12.2 g·kg^-1^, TC 737.4 g·kg^-1^ (with the C/N ratio 60.4), TP 9.51 g·kg^-1^, TK 14.6 g·kg^-1^, and specific surface area 41.9 m^2^·g^-1^.

### 2.3 Field experimental design and crop management

Rice (*var. Xiangzaoxian 45)* seedlings were transplanted in the last week of April and harvested in mid-July of 2023. Rice seedlings (25 days old) were transplanted at a spacing of 15.0 cm × 30.0 cm. The experiment comprised four treatments, which were arranged in a randomized complete block design with three replications: (i) N fertilizer alone (CK), (ii) N fertilizer + RSB (B), (iii) N fertilizer + MV (M), and (iv) N fertilizer + RSB + MV (BM). Each experimental plot had an area of 20 m^2^ (4 m × 5 m) and was separated by a ridge (0.45 m wide and 20 cm aboveground) to prevent the movement of water and nutrients between plots. Chemical fertilizers were applied to the field at the following rates: 150 kg N ha^-1^ (urea 46.4% N), 75 kg P ha^-1^ (super phosphate 12.0% P_2_O_5_), and 120 kg K ha^-1^ (potassium chloride 60% K_2_O). Urea was applied in three splits: 40% as a basal application, 30% as a top-dressing at the tillering stage, and another 30% as a top-dressing at the panicle initiation stage. Basal P and K fertilizers were consistently applied at the recommended rates.

MV was mechanically incorporated *in situ* annually at a rate of 22.5 t·ha^-1^ at a soil depth of ~18 cm at the full-bloom stage, 5 days before transplanting rice seedlings, and then flooded up to ~5 cm in depth. The RSB was applied simultaneously at a rate of 4 t·ha^-^¹ on the same day as MV, which was determined based on the annual return of biochar derived from the full amount of rice straw produced within a double-season rice system, thereby ensuring efficient recycling of field-derived biomass resources. The rice straw was removed annually from the plots after harvesting rice plants.

### 2.4 Soil sample and grain collections and determination of chemical characteristics

After harvesting rice plants in 2023, all grains in each plot were weighed to calculate the rice yield. Five soil cores were collected following a five-point sampling method from the plough layer (0–20 cm) of each replicate using a soil auger. The soil samples were then divided into three parts: one part was stored at 4 °C for determination of soil enzyme activities, NH_4_^+^, NO_3_^−^, MBC, and MBN; another part was stored at -80 °C for DNA extraction and further microbial analysis; and the rest was naturally air-dried and sieved to assess SOC and TN. At the maturity stage, rice plants were manually harvested from each replicate at the ground level. Grain yields were obtained by direct weighing after the crop was threshed. The grains were ground and sieved for preservation and subsequent analysis of the N content.

The content of SOC and TN was determined by the potassium dichromate (K_2_Cr_2_O7)-H_2_SO_4_ external heating method and Kjeldahl method, respectively. NH_4_^+^ and NO_3_^−^ were extracted with 2 mol·L^-1^ KCl, and the content of NH_4_^+^ and NO_3_^−^ was then determined using the indigo phenol blue colorimetry method and ultraviolet spectroscopy, respectively (Bao, 2000). Microbial biomass C and N (MBC and MBN) were extracted using the chloroform fumigation-K_2_SO_4_ extraction method (Brookes et al., 1985; Vance et al., 1987). Urease (*Ure*) activity was measured using the phenol-sodium hypochlorite colorimetric method, which was expressed in milligrams of NH_4_^+^ produced in 1 g of soil after 1 d of incubation (Guan, 1986). Nitrate reductase (*Nar*) activity was determined by the phenol disulfonic acid colorimetric method, and nitrite reductase (*Nir*) activity was determined by the α-naphthylamine sulfanilic acid colorimetric method (Kandeler et al., 1994). The N content of rice grains was measured using 5 mL each of H_2_O_2_ and H_2_SO_4_ at 120 °C (Bao, 2000).

### 2.5 DNA extraction and high-throughput Illumina sequencing and functional genes of N-cycling

Total genomic DNA was isolated from 0.5 g soil samples using a Fast DNA SPIN Kit (MagaBio Soil Genomic DNA Purification Kit). The purity and concentration of DNA were detected using a NanoDrop One spectrophotometer (Thermo Fisher Scientific, Waltham, USA). Amplification of the V4 region of the bacterial 16S rRNA gene with the primers 515F (GTGCCA GCMGCCGCGGTAA) and 806R (GGACTACHVGGGTWTCTAAT) was performed using the NEBNext^®^ Ultra™ DNA Library Prep Kit for Illumina (New England Biolabs, Ipswich, MA, USA) to generate the sequencing library. Finally, the library was sequenced on an Illumina Nova 6000 platform, and 250 bp paired-end reads were generated.

Functional genes related to N-cycling were detected using a high-throughput quantitative PCR-based chip. High-throughput, chip-based quantitative PCR was performed using the Magigene Biotechnology PCR system (Guangdong Magigene Biotechnology Co., Ltd.). Information regarding primers is provided in **Supplementary Table 1**. We mainly focused on N-cycling functional genes. After DNA extraction, the amount and purity of DNA were determined using a Qubit 2.0 instrument (Thermo Fisher Scientific, Waltham, USA). The qualified DNA samples were added to a 384-well microplate designated as the sample source plate, while primers and qPCR reagents were added to another 384-well microplate designated as the assay source plate. The reagents of both the sample source plate and assay source plate were dispensed into the wells of the SmartChip high-throughput qPCR system using the SmartChip Multisample Nanodispenser, a high-throughput automatic microsampling device (WaferGen Biosystems, USA). qPCR and fluorescence signal detection were performed using the SmartChip Real-time PCR System, and the amplification curves and dissolution curves were automatically generated. Quality control was conducted according to the cycle threshold (Ct) summary table derived from the Ct values provided by the SmartChip Real-time PCR System. Absolute quantitative information on the 16S rRNA gene was obtained through fluorescence qPCR (Roche, LightCycler 480). The absolute abundance of each target gene in each sample was then calculated based on the absolute quantity of the 16S rRNA gene.

### 2.6 Bioinformatics analysis and statistical analysis

Raw sequences were processed with QIIME, and chimeras were detected using UCHIME. After quality filtering and removal of chimeric sequences, all remaining high-quality sequences were clustered into operational taxonomic units (OTUs) at 97% similarity using Usearch software (version 10.0, http://www.drive5.com/usearch). BLAST was used to taxonomically annotate the representative sequences. Alpha diversity indices were generated based on the obtained OTUs.

IBM SPSS Statistics 26.0 was used to analyze the data. A two-way analysis of variance (ANOVA) and multiple comparisons based on the least significant difference (LSD) were used to assess the significance of differences (*P* < 0.05) in N accumulation, rice yield, content of soil nutrients, enzyme activities, and abundances of N-cycling functional genes. Furthermore, the redundancy analysis (RDA) of Canoco 5.0 software was used to analyze the relationship between the chemical properties and microbial communities. The figures were created using Origin 9.8 (Origin Lab).

Biological statistical analyses were constructed and visualized using R software (version 4.3.3). Specifically, diversity index values, including microbial richness (Chao1 index) and diversity (Shannon index), were calculated based on ASV tables using the “vegan” package. In addition, microbial β-diversity was estimated based on Bray-Curtis distances through constrained principal component analysis (CPCoA) using the “amplicon” package to visualize variation in microbial community structure and calculate relative abundances using the “plyr” package. Mantel analysis was performed using the “dplyr” and “ggcor” packages. Linear regression analyses were used to test the significance of statistical associations. Using the “plspm” package for partial least squares path modeling (PLS-PM) analysis. The overall fit of the PLS-PM was evaluated using the goodness-of-fit index.

## 3 Results

### 3.1 Impact of RSB and MV on N accumulation and rice grain yield

Application of RSB and MV either alone (B and M) or in combination (BM) promoted N accumulation and rice grain yield (**Supplementary Figure 2**). N accumulation was 6.09% and 36.52% higher and yield was 2.77% and 6.13% higher in the B and M plots than in the CK plots, respectively; N accumulation and yield were 40.72% and 14.43% higher in the BM plot than in the CK plot, respectively.

### 3.2 Impact of RSB and MV on soil C and N levels and enzymatic activities

The combination of RSB with MV significantly increased the soil C and N content (**Table 1**). The SOC was 9.89%, 10.24%, and 23.82% higher in the B, M, and BM plots than in the CK plots, respectively. TN was 14.95% higher in the BM plot than in the CK plots (*P* < 0.05). The NH_4_^+^ and NO_3_^−^ content was 17.42–26.52% and 24.26–35.98% higher in the B, M, and BM treatments than in the CK, respectively (*P* < 0.05). MBC was 16.63%, 13.75%, and 35.20% higher and MBN was 3.99%, 2.68%, and 14.27% higher in the B, M, and BM plots than in the CK plots, respectively (*P* < 0.05). The MBC/MBN ratio was 1.85%, 0.34%, and 7.73% higher and *q*MB was 13.43%, 9.70%, and 16.42% higher in the B, M, and BM plots than in the CK plots, respectively. Furthermore, the activities of *Ure*, *Nar*, and *Nir* were significantly increased when RSB and MV were applied (**Supplementary Figure 3**). The *Ure* activity was 28.03%, 10.62%, and 15.92% higher; *Nar* activity was 27.19%, 25.44%, and 88.60% higher, and *Nir* activity was 30.77%, 72.93%, and 53.56% higher in the B, M, and BM treatments, respectively, than in the CK (*P* < 0.05).

**Table 1:** Effects of RSB and MV on C and N contents of reddish paddy soil and two-way ANOVA results.

Treatments	SOC (g·kg^-1^)	TN (g·kg^-1^)	NO_3_^−^ (mg·kg^-1^)	NH_4_^+^ (mg·kg^-1^)	MBC (mg·kg^-1^)	MBN (mg·kg^-1^)	MBC/MBN	qMB
CK	18.16 ± 0.20b	1.13 ± 0.04b	1.32 ± 0.17b	19.04 ± 1.26c	244.21 ± 0.55c	45.35 ± 0.03b	5.95 ± 0.03b	1.34 ± 0.19ab
B	18.78 ± 0.37b	1.15 ± 0.04ab	1.58 ± 0.20a	24.36 ± 1.82ab	284.83 ± 4.29b	47.16 ± 3.33b	6.06 ± 0.49ab	1.52 ± 0.02b
M	18.84 ± 0.08b	1.11 ± 0.01b	1.55 ± 0.30a	23.66 ± 0.99b	277.78 ± 12.91b	46.57 ± 0.96b	5.97 ± 0.40ab	1.47 ± 0.07b
BM	21.16 ± 0.15a	1.23 ± 0.05a	1.67 ± 0.15a	25.89 ± 1.04a	330.16 ± 19.64a	51.82 ± 4.35a	6.41 ± 0.31a	1.56 ± 0.15a
B	48.7^***^	12.4^**^	10.8^**^	52.3^***^	61.5^***^	7.3^*^	5.6^*^	9.8^**^
M	9.2^**^	3.7^ns^	12.4^**^	11.6^**^	5.1^*^	4.8^*^	0.6^ns^	4.9^*^
B×M	15.4^***^	6.2^*^	0.4^ns^	16.3^***^	6.8^*^	2.7^ns^	1.3^ns^	5.2^*^

SOC, soil organic C; TN, total N; NO_3_^−^, nitrate N; NH_4_^+^: ammonium N; MBC, microbial biomass of C; MBN, microbial biomass of N; qMB, microbial entropy. Different letters denote significant difference at *P* < 0.05. The values represented by the rows labeled “B”, “M”, and “B × M” are F-values from the two-way ANOVA. Significance levels in ANOVA are indicated as: ***P < 0.001, **P < 0.01, *P < 0.05, and ns indicates no significant difference (*P* > 0.05).

### 3.3 Impact of RSB and MV on soil microbial diversities, community composition, and their correlation with soil environmental factors

**Figure 1:**
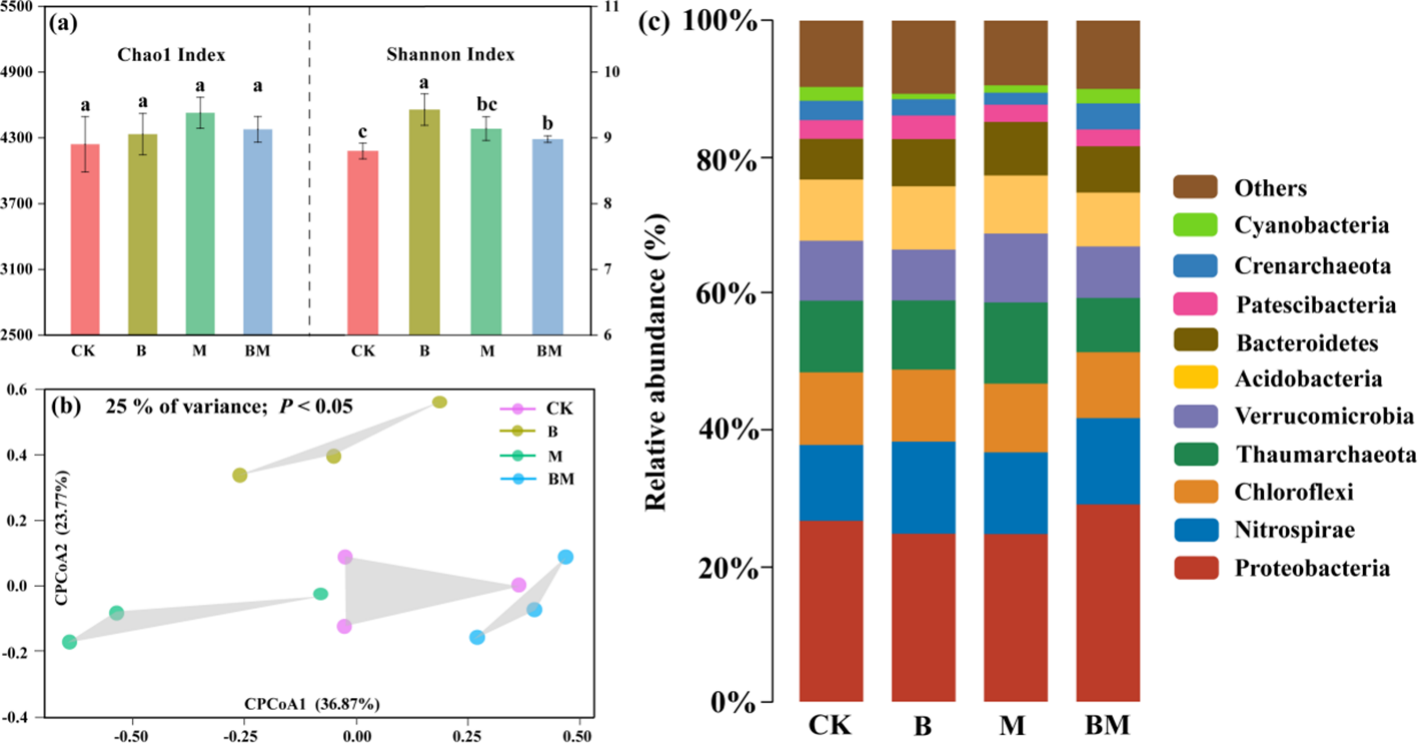
Effects of RSB and MV on α-diversity (Chao1 and Shannon index) of bacterial community **(a)**, the constrained principal component analysis (CPCoA) for β-diversity of bacterial community based on 16S-rRNA gene **(b)**. Statistically significant differences among treatments are represented by different letters (*P* < 0.05). Soil bacterial community compositions (Phylum level) among different experimental groups and the relative abundance of bacterial phyla **(C)**. “Other” refers to all other taxa with an abundance lower than 0.9%.

Application of RSB and MV alone or in combination improved the structure and diversity of the soil bacterial community (**Figure 1**). The most abundant phyla were *Proteobacteria*, *Nitrospirae*, *Chloroflexi*, *Thaumarchaeota*, *Verrucomicrobia*, *Acidobacteria*, and *Bacteroidetes*, which accounted for more than 80% of the bacterial sequences (**Figure 1a**). In addition, the relative abundances of C and N-cycling-associated microbes such as *Proteobacteria*, *Nitrospirae*, and *Bacteroidetes* were increased in the BM treatment, and the relative abundances of *Thaumarchaeota*, *Verrucomicrobia*, and *Acidobacteria* were reduced.

Soil bacterial species richness and the Chao1 index were higher in the B, M, and BM treatments than in the CK (*P* > 0.05); compared to CK plots, the B and BM treatments markedly increased the Shannon indices by 7.16% and 2.05%, respectively (**Figure 1b**; *P* < 0.05). Conversely, no significant differences (*P* > 0.05) in the β-diversity of the soil bacterial community were observed when MV was applied alone or in combination with RSB (M and BM); however, greater differentiation in the β-diversity of the soil bacterial community was observed when RSB was applied alone (B) (**Figure 1c**; *P* < 0.05).

**Figure 2:**
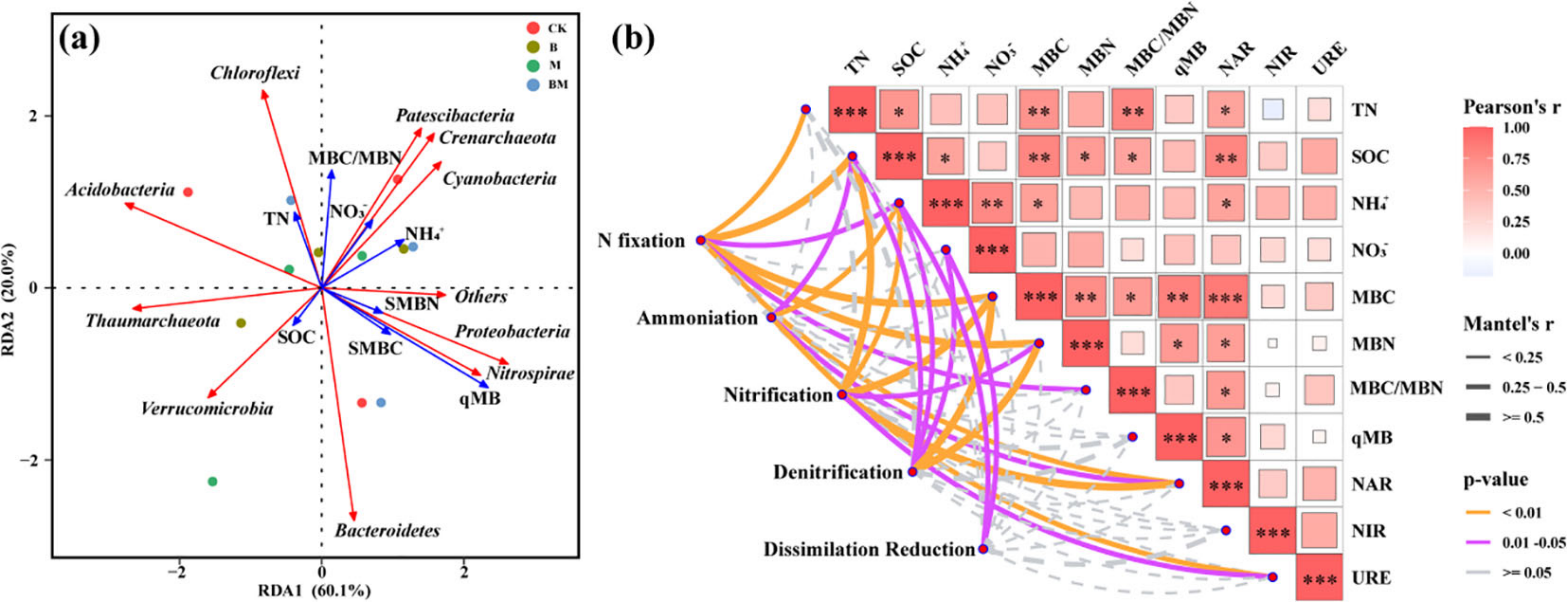
Redundancy analysis (RDA) of soil microbial community structure and diversity with soil environment factors **(a)** the Mantel test between the functional genes related to N-cycling, C and N contents, and soil enzymatic activities **(b)**. RDA analysis shows an association between soil bacterial community and environmental factors. Circles represent the microbial community at different treatments (at the phylum level), while arrows represent environmental measurement parameters. The Mantel test revealed the association between N-cycling microbial functional genes and soil nutrients and enzyme activities, with the heatmap illustrating the pairwise comparisons of soil nutrients and enzyme activities through Pearson correlation analysis. *** represents P < 0.001, ** represents P < 0.01 and * represents P < 0.05.

RDA revealed that RSB and MV altered the composition of the bacterial community at the phylum level by affecting soil environmental variables, including soil TN, SOC, NO_3_^−^, NH_4_^+^, MBC, MBN, MBC/MBN, and *q*MB (**Figure 2a**). The two ordination axes explained 60.1% and 20.0% of the variation in bacterial community structure. Among the soil environmental factors, *q*MB, MBC/MBN, and the NH_4_^+^ content had relatively stronger effects on bacterial community composition, whereas NO_3_^−^ and SOC had relatively weaker effects. Mantel analysis showed that the abundance of a functional gene (*nif*H) related to N-fixing microbes was strongly positively correlated with TN, SOC, NH4^+^, MBC, MBN, Nar, and Ure (**Figure 2b**). Abundances of functional genes (*ure*C, *gdh*A) responsible for ammonification were also strongly positively correlated with SOC, NH_4_^+^, and Nar. The abundances of functional genes related to nitrifiers (*amo*A) were strongly positively correlated with SOC, NH_4_^+^, NO_3_^−^, MBC, MBN, *Ure*, and *Nar*, while the abundances of functional genes related to denitrifiers (*amo*A, *nar*G, *nir*S, *nir*K, and *nos*Z) were significantly positively correlated with SOC, NH_4_^+^, NO_3_^−^, MBC, MBN (*P* < 0.05 or 0.01). The abundance of functional genes (*nap*A) related to dissimilatory reduction was strongly positively correlated with NH_4_^+^ and NO_3_^−^ (*P* < 0.05).

### 3.4 Impact of RSB and MV on the abundance of soil N-cycling functional genes

**Figure 3:**
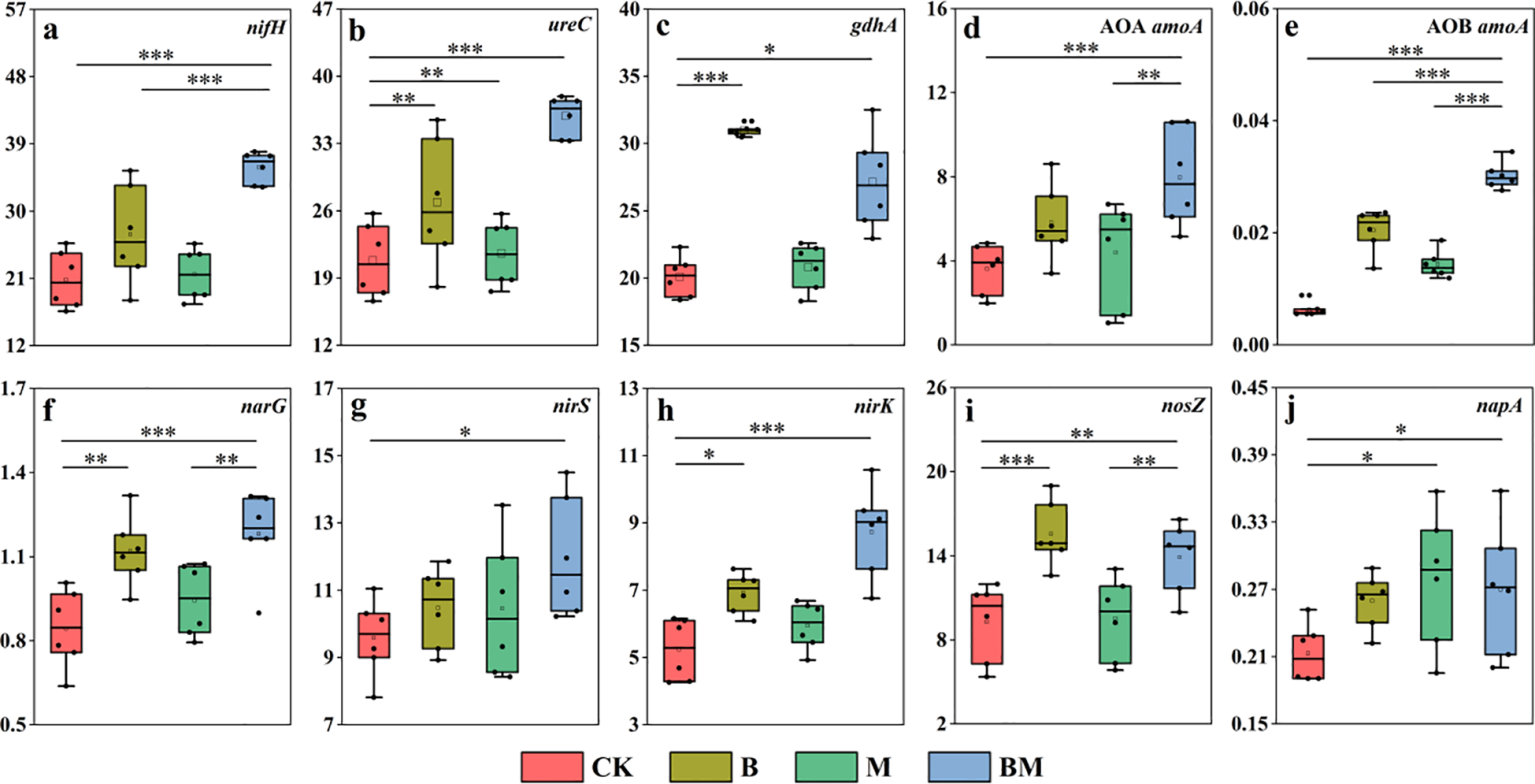
Effects of applying RSB and MV on abundances of AOA *amo*A **(a)**, AOB *amo*A **(b)**, *nar*G **(c)**, *nap*A **(d)**, *nir*S **(e)**, *nir*K **(f)**, *nos*Z **(g)**, *nif*H **(h)**, *ure*C **(i)** and *gdh*A **(j)** (n=6). Horizontal lines indicate pairwise comparisons between the two connected treatments. Statistically significant differences among experimental groups are indicated by asterisks, with ***P < 0.001, **P < 0.01 and *P < 0.05.

Application of RSB and MV, either individually or in combination, had a positive effect on the abundances of functional genes associated with N-fixation and ammonification in reddish paddy soil (**Figures 3a–c**). The abundances of the *nif*H, *ure*C, and *ghd*A genes were 29.09% and 72.25%, 40.02% and 49.32%, and 54.13% and 34.98% higher in the B and BM treatments than in the CK, respectively (*P* < 0.05).

The application of RSB and MV also significantly enhanced the abundance of functional genes related to nitrification in paddy soil (**Figures 3d, e**). Specifically, the abundances of the AOA *amo*A and AOB *amo*A genes were 60.96% and 21.61% higher in the B plot, 120.50% and 233.33% higher in the M plot, and 133.33% and 400.00% higher in the BM plot than in the CK, respectively.

The abundance of *nar*G was highest in the BM treatment, and it was 40.48% higher in the BM treatment than in the CK; the abundance of *nar*G was also higher in the B and M treatments than in the CK, albeit these differences were not significant (**Figure 3f**; *P* < 0.05). The abundances of the *nir*S and *nir*K genes were consistently higher in the B, M, and BM treatments than in the CK; the abundance of *nir*S was 9.18%, 9.07%, and 24.71% higher and that of *nir*K was 13.58%, 30.08%, and 66.92% higher in the B, M, and BM treatments than in the CK, respectively (**Figures 3g, h**). Similarly, the abundance of the *nos*Z gene was 67.49% and 49.52% higher in the B and BM treatments than in the CK, respectively (**Figure 3i**); the abundance of the *nap*A gene involved in dissimilatory nitrate reduction to ammonium (DNRA) was 23.81%, 33.33%, and 28.57% higher in the B, M, and BM treatments than in the CK, respectively (**Figure 3j**; *P* < 0.05).

**Figure 4:**
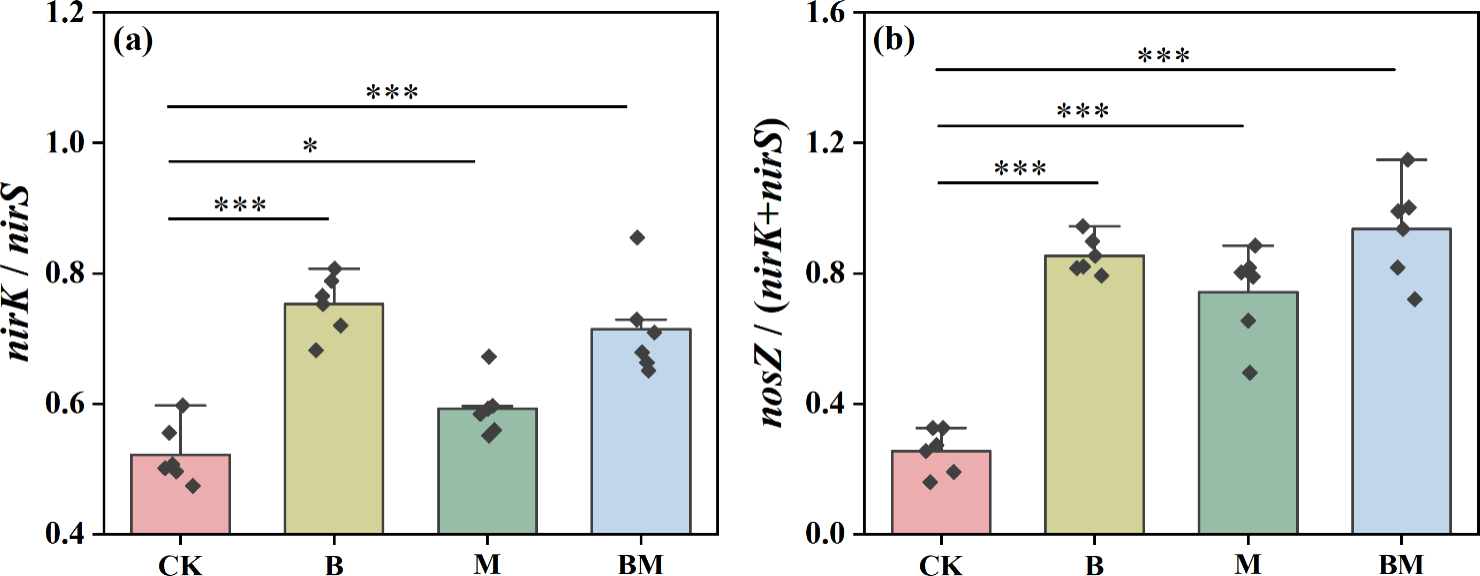
The effects of applying RSB and MV on ratios of *nir*K/*nir*S **(a)** and *nos*Z/(*nir*S+*nir*K) **(b)** (n=6). The horizontal line within each violin represents the median. Horizontal lines indicate pairwise comparisons between the two connected treatments. Statistically significant differences among experimental groups are indicated by asterisks, with ***P < 0.001 and *P < 0.05.

Application of RSB and MV significantly altered the composition of denitrifiers (**Figure 4**). The *nir*K/*nir*S ratio was 44.23%, 13.46%, and 36.54% higher, and that of *nos*Z/(*nir*S+*nir*K) was 226.92%, 184.62%, and 261.54% higher in the B, M, and BM treatments than in the CK, respectively (*P* < 0.05).

### 3.5 Relationship of N accumulation and rice yield with the abundance of soil functional genes related to nitrifiers and denitrifiers

Linear regression analysis revealed a significant positive correlation of N accumulation and rice grain yield with the abundances of functional genes (AOA *amo*A, AOB *amo*A, *nar*G, *nir*S, *nir*K, and *nos*Z) related to nitrifiers and denitrifiers in reddish paddy soil (**Supplementary Figure 4**; *P* < 0.05).

### 3.6 The PLS-PM relationships of N accumulation and rice yield with soil nutrients, microbial community, and the abundance of N-cycling functional genes

**Figure 5:**
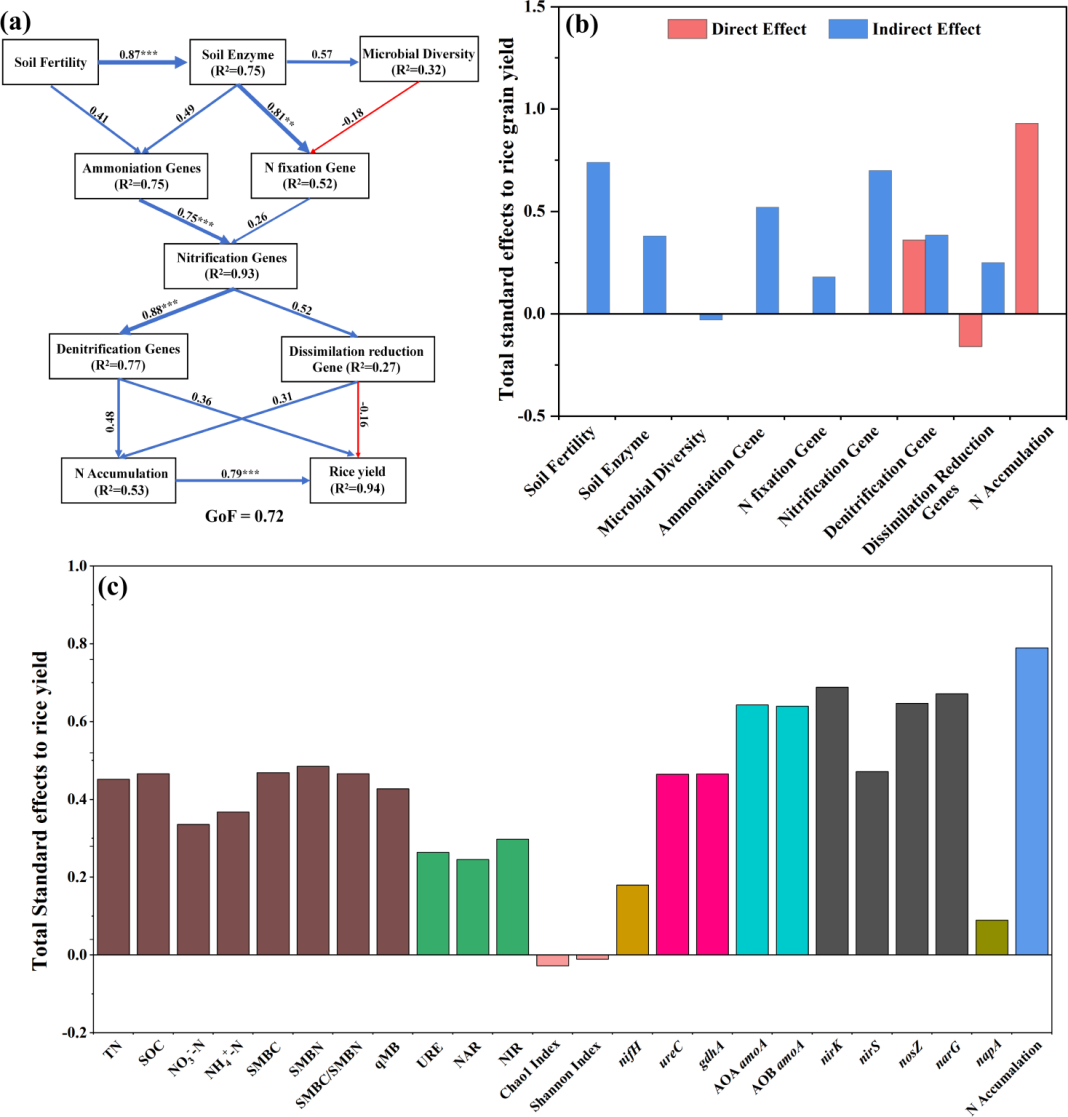
PLS-PM model describing the effects of contents of C and N of soil, enzyme activities, microbial community diversity, and functional gene abundances related to N-cycling microbes on N accumulation and rice yield **(a)**, and standardize these variables for their direct and indirect effects **(b)**, as well as the cross-loading effects of each observed variable on rice yield **(c)**. Path coefficients are labeled beside the arrow lines. Solid and dashed arrows indicate direct and indirect effects, while red and blue arrows indicate positive and negative effects, respectively. Statistically significant differences among experimental groups are indicated by asterisks, with *** *P* < 0.001, ** *P* < 0.01, * *P* < 0.05. The R2 represents the variance of the variables considered by the model. The goodness of fit (GoF) is used to evaluate the model, with a GoF value of 0.72.

PLS-PM was performed to elucidate the complex relationships between soil nutrients and microbial characteristics (**Figure 5a**). The model revealed that soil nutrients not only had an extremely significant positive effect on enzyme activity (path coefficient of 0.87, *P* < 0.001) but also directly and regulated the abundance of N-ammonifying functional genes (path coefficient of 0.41). Soil enzyme activity was strongly positively correlated with N-fixation functional genes (path coefficient of 0.81, *P* < 0.01). Soil N-ammonification genes have a positive effect on N-nitrifying genes (path coefficient of 0.75, *P* < 0.001), while N-nitrifying genes have a positive effect on N-denitrifying and gene involved in DNRA (path coefficient of 0.88 and 0.52). A negative correlation was observed between genes associated with N-fixation and microbial community, though the effect was not significant. N accumulation was a direct positive driver of yield increases (path coefficient of 0.79, *P* < 0.001).

Furthermore, standardized path analysis revealed that soil nutrients and N accumulation had the strongest direct positive effect on rice yield (with the highest standardized effect of 0.74 and 0.93, respectively), followed by N-nitrifying and -ammonifying microbial functional genes (standardized effect of 0.70 and 0.52, respectively), which increased rice yield. Conversely, DNRA genes had the strongest negative effect on rice yield (**Figure 5b**). Cross-loading analysis suggested that TN, SOC, MBC, MBN, the MBC/MBN ratio, enzyme activities, and the abundance of *nifH*, *gdhA*, *ureC*, *nirK*, *narG* significantly positively affected rice yield (**Figure 5c**). Overall, soil nutrients, the abundance of N-denitrifying functional genes, and N accumulation were the main drivers of increases in rice yield.

## 4 Discussion

### 4.1 RSB and MV enhanced N accumulation and rice yield

We found that both RSB and MV enhanced N accumulation and rice yields, and the combination of RSB with MV had the strongest effect (**Supplementary Figure 2**). After being incorporated into the soil, organic materials (i.e. RSB and MV) undergo microbial decomposition, subsequently causing the release of various nutrients (i.e., C, N, P, and K) (Xia et al., 2024), eventually providing a balanced supply of nutrients for rice plants. Furthermore, the use of leguminous cover crops (e.g., MV) can increase the N content by symbiosis or stimulate autotrophic N fixation in (Chen et al., 2020). RSB adsorbs nutrients through its large specific surface area and abundant functional groups, providing more favorable conditions for microbial growth (Azeem et al., 2023). Both RSB and MV promote the absorption and accumulation of N, thereby enhancing the yield of rice plants.

Moreover, numerous studies have demonstrated the benefits of combining GM with rice straw or biochar for soil microbes, N characteristics, and crop performance (Cheng et al., 2023). Our previous studies have shown that RSB improves N compatibility in soil–rice plant systems by stimulating N release during the initial rapid phase of decomposition, which noticeably decreases the rates of N release when MV residues are incorporated into paddy soil (Xie et al., 2022). The combination of RSB with MV induces increased microbial diversity (**Figure 1**) and the abundance of N-cycling functional genes (**Figures 3**, **4**), further accelerating the N turnover rate (**Table 1**), ultimately enhancing N accumulation and rice yield. Although the Shannon index was higher in the B treatment than in BM, this does not contradict the synergistic effects observed in functional processes. Biochar may enhance overall microbial diversity by creating heterogeneous niches, whereas milk vetch may preferentially promote the proliferation of dominant microbial groups, for example by stimulating K-strategist microorganisms while suppressing the dominance of r-strategists (Li et al., 2026; Xu et al., 2023). Additionally, the abundance of functional genes associated with nitrifiers/denitrifiers was positively correlated with rice yields (**Supplementary Figure 4** and 5C). Additionally, the co-application of RSB and MV may have enhanced the leaf area index and photosynthetic efficiency (Islam et al., 2019). Therefore, the combination of RSB with MV prolongs N availability and ensures that N is released at times when it is most needed by rice plants, thereby optimizing the yield components of rice, ultimately leading to increased rice production (Liu et al., 2021).

### 4.2 Responses of soil C and N nutrients to RSB and MV

Soil fertility and health are closely linked to C and N dynamics (Zang et al., 2024). Previous studies have demonstrated the effectiveness of MV and RSB application for increasing soil C and N nutrients (Wang et al., 2023). Our results indicated that applying RSB and MV alone or in combination significantly enhanced the content of SOC, TN, NH_4_^+^, NO_3_^−^, MBC, and MBN as well as the MBC/MBN ratio and *q*MB by 14.3%–88.6% (**Table 1**). This enhancement can be attributed to the ability of RSB and MV to return substantial C and N to paddy soil (Cheng et al., 2023). For example, an increase in the soil TN content is related to the availability of N from RSB (12.1 g·kg^-1^) and MV residues (25.5 g·kg^-1^). The combination of RSB with MV resulted in a significant increase of 5.70% in soil C and 12.67% in the N content compared with the sole application of either amendment (**Table 1**). Similar results were obtained by Cheng et al. (2023) in a pot experiment. A likely explanation for the synergistic effect of their combined application may be that MV with a low C/N ratio (16.3) often results in high and rapid N mineralization, while RSB with a high C/N ratio (61.3) usually leads to low N mineralization and slow N release. Therefore, C and N losses were alleviated by the combination of RSB with MV.

The combination of RSB with MV also significantly enriched populations of soil microbes (e.g., *Proteobacteria* and *Bacteroidetes*), thereby promoting the degradation of SOM, in turn maintaining soil fertility (Qian et al., 2022). As a result, an increase in eutrophic microbial activity can also increase the available C and N content (Cheng et al., 2023); this was confirmed by the results of the RDA, revealing a strong relationship between soil nutrients and microbial community structure (**Figure 2**). This correlation might stem from the ability of RSB and MV to improve soil aeration and raise soil pH, thereby optimizing the soil micro-environment (Ren et al., 2021).

Furthermore, the NH_4_^+^ and NO_3_^−^ content was significantly higher in the BM treatment than in the CK (**Table 1**), leading to the enhanced abundance of N-cycling functional genes (especially *nif*H, *ure*C, and *gdh*A), as demonstrated by Mantel analyses (**Figure 2B**). The enhanced abundance of these functional genes accelerates the conversion of soil organic N to inorganic N and augments the availability of soil nutrients (Khan et al., 2020). Concurrently, the increased abundance of *ure*C (**Figure 3b**) in the BM treatment is consistent with the increase in Ure activity (**Supplementary Figure 3A**), providing indirect evidence for the positive effect of RSB and MV on soil NH_4_^+^ content. The qMB, a critical indicator of soil health, was significantly higher in BM treatment compared with CK, indicating that soil amendments with organic materials can increase microbial efficiency via the conversion of SOC into MBC and promote C stability (Massaccesi et al., 2024).

### 4.3 RSB and MV regulated N-cycling related soil microbial functions

The N-cycling process, primarily driven by soil microbes through nitrification and denitrification, has a major impact on the soil N supply and environmental risks. Our results indicated that the abundance of functional genes associated with N-fixing, ammonification, nitrification, denitrification, and dissimilatory reduction increased in the BM treatment (**Figure 3**). Overall, MV and RSB promote the growth of soil microbes by increasing soil nutrients, in turn enhancing their N-fixation, nitrification, and denitrification capabilities to regulate soil N availability (Zheng et al., 2024).

The PLS-PM analysis provided evidence of the relationships among N-cycling-related functional genes (e.g. N-fixation, ammonification, nitrification, denitrification), soil fertility, and enzymes (**Figure 5a**). We found that N-cycling genes (especially *nif*H) were closely related to soil biotic factors such as MBC and MBN (**Figure 2b**). Such effects on N fixation genes likely stemmed from the ample C and N pools for microbes, promoting the reproduction of N-fixing bacteria (Liao et al., 2023). The enhanced activities of enzymes might mediate soil N conversion (Zumstein and Helbling, 2019), especially Nar and Nir genes, which play a key role in the reduction of nitrate to nitrite. Additionally, increased Ure activity contributes to NH_4_^+^ accumulation and provides additional substrates for the nitrification process. Activities of all these enzymes were enhanced by RSB and MV, thus promoting the nitrification/denitrification process (He et al., 2023).

Moreover, shifts in the abundance of functionally important microbial groups likely have key implications for soil N-cycling process (Frey et al., 2023). RSB and MV affected the dynamics of microbe-mediated N-cycling by altering the abundance of functional genes (**Figure 3**). For example, RSB and MV may disrupt soil N-cycling by increasing the abundance of AOA *amo*A and AOB *amo*A, which may enhance ammonia oxidation, and subsequently modulate N availability, thus leading to changes in other N-cycling processes (Yang et al., 2018). Additionally, a tight linkage between N-fixing microbes and nitrifiers has been previously reported (Li et al., 2020). Therefore, RSB and MV may enhance the diversity of N-fixing bacteria, thus influencing soil N-fixation process and altering the NH_4_^+^/NO_3_^−^ ratio, indirectly regulating nitrification and denitrification processes (Espenberg et al., 2018).

Another interesting observation is that the abundances of the *nir*S and *nir*K genes were higher in BM treatment than in the B and M treatments, which likely stems from the increased inorganic N (**Figures 3g, h**). This is consistent with the finding that a higher NH_4_^+^ concentration increased the abundances of microbes containing *nir*K or *nir*S genes, as a higher NH_4_^+^ concentration can increase the supply of NO_3_^−^, which provides a direct substrate for the growth of denitrifiers (Yi et al., 2015). Yuan et al. (2022) showed that biochar amendment is an effective strategy for stimulating the DNRA process in paddy soil, which is consistent with the results of our findings (**Figure 3j**). In addition, the stimulation of the DNRA process in the BM treatment may be attributed to biochar-induced changes in soil microenvironment (Zhou et al., 2019b). The porous structure of biochar can promote soil aggregation and create microsites with limited oxygen diffusion, favoring DNRA microorganisms under low-redox conditions (Wei et al., 2022). Such microenvironmental heterogeneity may shift nitrogen transformation pathways toward nitrate reduction to ammonium (Kraft et al., 2014).

The application of RSB and MV resulted in significant changes in the structure of the denitrifying microbial community. The synergistic effect of the RSB and MSV on the ratio of *nos*Z/(*nir*K+*nir*S) was found to be greater than that of the sole application of either RSB or MSV (**Figure 4**). Qiu et al. (2024) revealed a positive correlation between N_2_O emissions and the composition of denitrifying microbial communities. Therefore, the combination of RSB with MV facilitates the reduction of N_2_O to N_2_, which is crucial for mitigating GHG emissions from reddish paddy fields. This further highlights the pivotal role of the combination of RSB with MV in promoting rice production in a sustainable manner, improving soil fertility, and lowering eco-environmental costs. However, our field experiment did not cover a longer time scale (e.g., > 10 yr) responses of greenhouse gases (e.g., N_2_O) to the co-addition of RSB and MV. Additionally, it is crucial to assure soil N retention in the waterlogged paddy fields. Thus, future studies should focus on assessing the impact of co-addition of RSB and MV on N_2_O emission, soil N retention and nitrate leaching in reddish paddy fields through field location experiments, enabling a more comprehensive evaluation of its environmental and ecological effects.

## 5 Conclusions

Applying RSB and MV, alone or in combination, improved soil N availability by optimizing microbial functions in paddy fields. Notably, co-applying RSB and MV markedly enhanced the enzyme activities and abundances of functional genes related to N-cycling, and further facilitated N absorption and yield increases of rice grain. Therefore, combining RSB with GM is a feasible strategy for promoting soil fertility, rice productivity and N_2_O mitigation potential. Moreover, future studies should further focus on the carbon and nitrogen footprints, as well as the economic benefits of rice production in rice-based cropping systems under combination of RSB with MV.

## Data Availability

The datasets generated and/or analyzed during the current study are available from the corresponding author on reasonable request.
