# Correction: Biochar and milk vetch synergistically enhance rice yield and soil fertility via regulating N-cycling in reddish paddy fields

**DOI:** 10.3389/fpls.2026.1921918

**Published:** 2026-07-29

**Authors:** Jiahui Zhou, Kun Zhang, Farooq Shah, Su Shiung Lam, Zhijian Xie

**Affiliations:** 1 College of Land Resources and Environment, Jiangxi Agricultural University, Nanchang, China; 2 Jiangxi Institute of Red Soil and Germplasm Resources, Nanchang, China; 3 Department of Agronomy, Abdul Wali Khan University Mardan, Khyber, Pakhtunkhwa, Pakistan; 4 Higher Institution Centre of Excellence (HICoE), Institute of Tropical Aquaculture and Fisheries (AKUATROP), Universiti Malaysia Terengganu, Kuala Nerus, Terengganu, Malaysia; 5 University Centre for Research and Development, Department of Chemistry, Chandigarh University, Mohali, Punjab, India

**Keywords:** microbial functions, milk vetch, rice productivity, rice straw biochar, soil N availability

The funder “the Technical Research Project of China National Tobacco Corporation Jiangxi Branch”, “202201001” to “Zhijian Xie” was erroneously omitted from the **Funding** statement.

The original version of this article has been updated.

