# Supplementary material for: Biochar and milk vetch synergistically enhance rice yield and soil fertility via regulating N-cycling in reddish paddy fields
Source: Front Plant Sci. 2026 Jun 19;17:1839609. doi: 10.3389/fpls.2026.1839609 (PMC13328393; doi:10.3389/fpls.2026.1839609)
Supplement: Supplementary file 1 [file Table1.docx]

**Appendix A. Supplementary material**


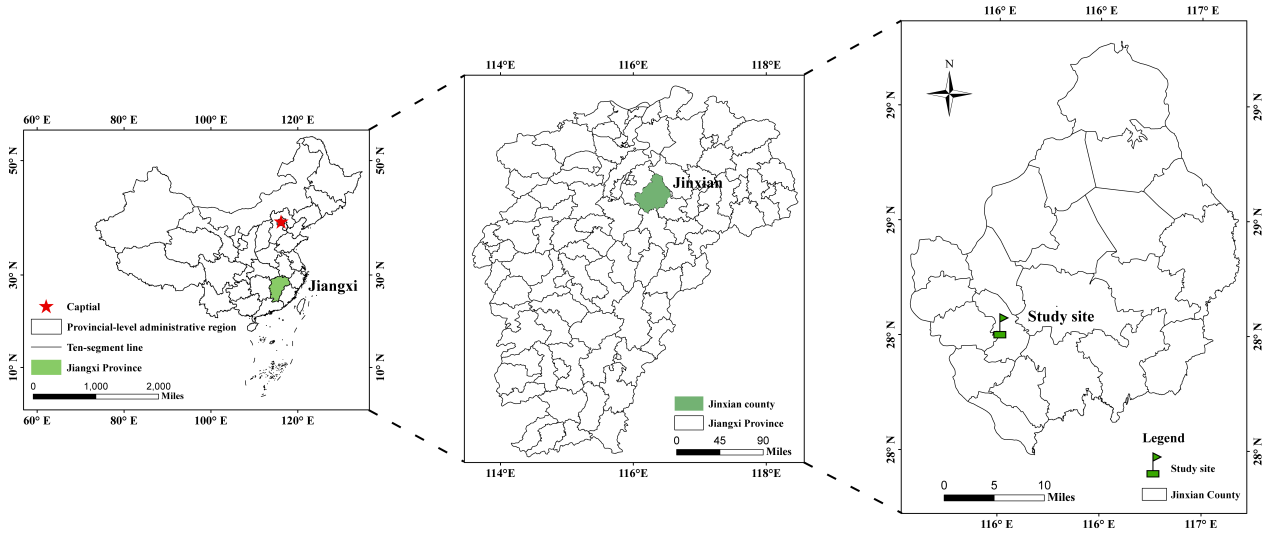


**Figure S1.** Map location of the study area.


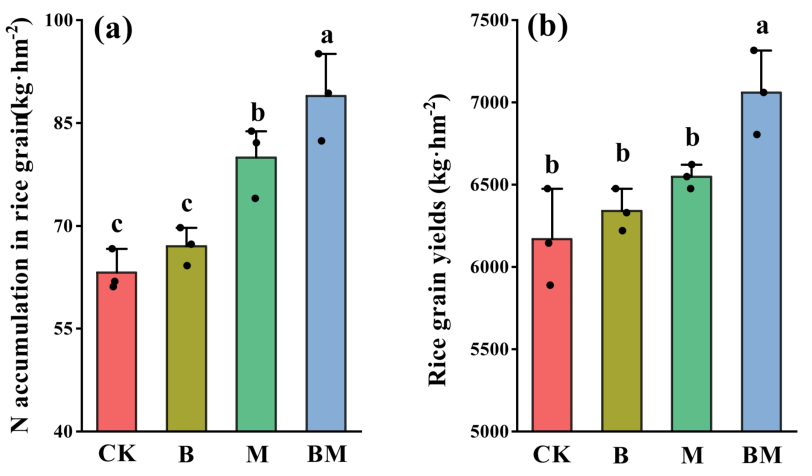


**Figure S2.** Effects of RSB and MV on N accumulation (a) and yield of rice grain (b). Statistically significant differences among treatments are represented by different letters (*P* < 0.05).


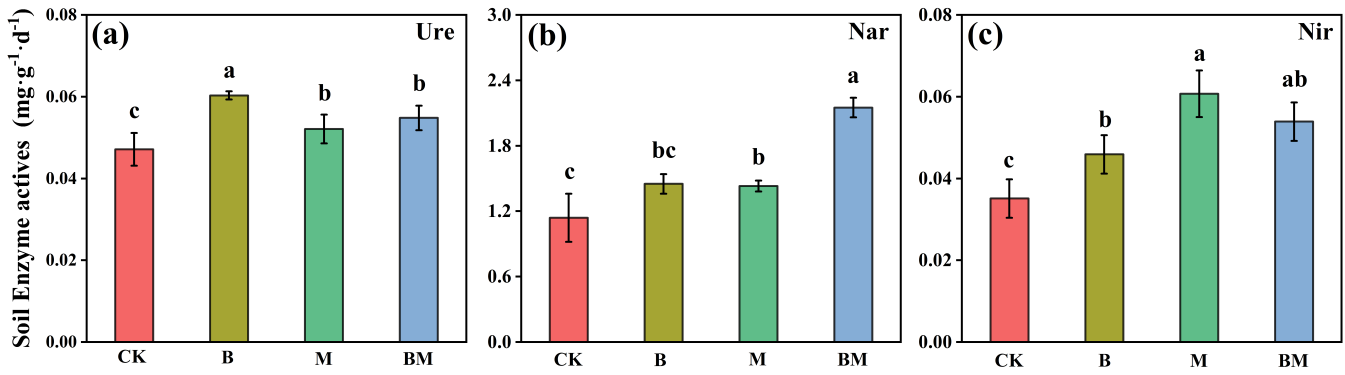


**Figure S3.** Effects of RSB and MV on soil *Ure* (a), *Nar* (b), *Nir* (c). Urease (*Ure*), nitrate reductase (*Nar*), and nitrite reductase (*Nir*). Statistically significant differences among treatments are represented by different letters (*P* < 0.05).

**
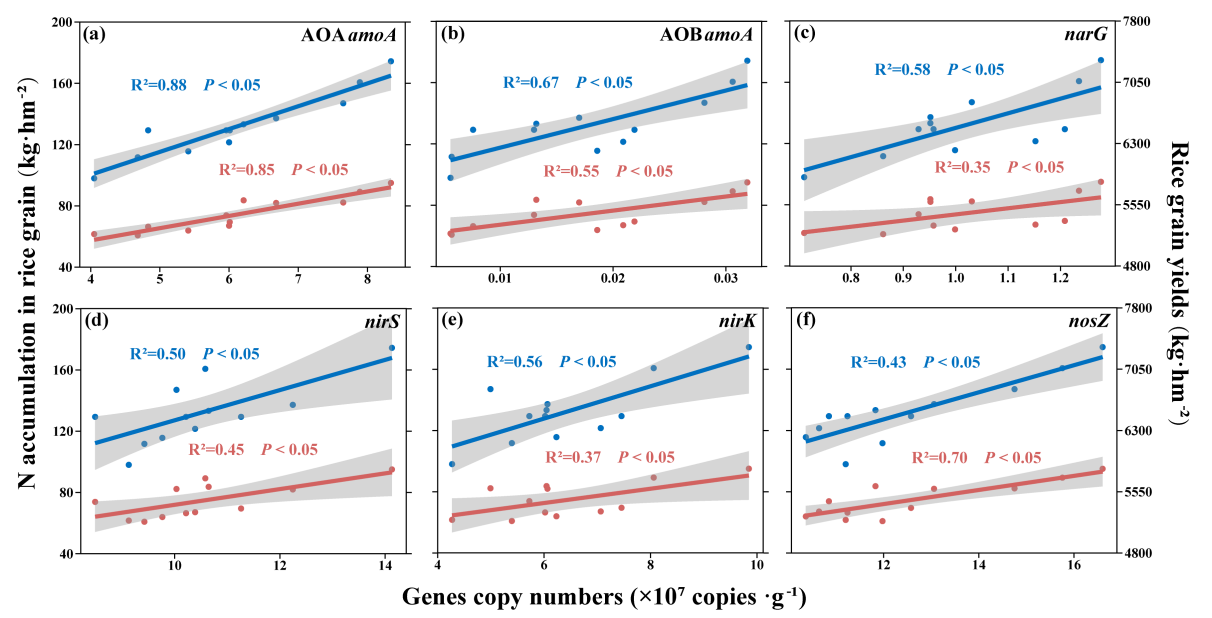
**

**Figure S4.** The linear regression relationships between the abundances of AOA *amo*A (a), AOB *amo*A (b), *nar*G (c), *nir*S (d), *nir*K (e), *nos*Z (f) and N accumulation (red), yield (blue) of rice grain. Solid lines indicate the predicted relationships are significant (*P* < 0.05) based on linear regression estimated using ordinary least squares. The shaded region represents the 95% confidence interval for the regression lines.
