# Supplementary material for: Biochar and milk vetch synergistically enhance rice yield and soil fertility via regulating N-cycling in reddish paddy fields
Source: Front Plant Sci. 2026 Jun 19;17:1839609. doi: 10.3389/fpls.2026.1839609 (PMC13328393; doi:10.3389/fpls.2026.1839609)
Supplement: Supplementary file 2 [file Table2.docx]

**Appendix B. Supplementary material**

**Table S1**

Primer information for N-cycling functional genes

| Gene names | Function | Forward sequence |
| --- | --- | --- |
| *nifH* | nitrogenase iron protein | AAAGGYGGWATCGGYAARTCCACCAC |
| *ureC* | urease | AAGMTSCACGAGGACTGGGG |
| *gdhA* | glutamate dehydrogenase | GCCATCGGYCCWTACAAGGG |
| AOA*-amoA* | ammonia monooxygenase α-subunit (Archaea) | STAATGGTCTGGCTTAGACG |
| AOB*-amoA* | ammonia monooxygenase α-subunit (Bacteria) | GGGGTTTCTACTGGTGGT |
| *narG* | nitrate reductase α chain | TAYGTSGGGCAGGARAAACTG |
| *nirS* | nitrite reductase (NO-forming) | GTSAACGTSAAGGARACSGG |
| *nirK* | nitrite reductase (NO-forming) | TGCACATCGCCAACGGNATGTWYGG |
| *nosZ* | nitrous-oxide reductase | CGCRACGGCAASAAGGTSMSSGT |
| *napA* | periplasmic nitrate reductase | CTGGACIATGGGYTTIAACCA |
